# Academic Burnout, Personality, and Academic Variables in University Students

**DOI:** 10.3390/ejihpe14060103

**Published:** 2024-06-03

**Authors:** Elena Cuevas-Caravaca, Elisa Isabel Sánchez-Romero, Josefa A. Antón-Ruiz

**Affiliations:** 1 Facultad de Educación, Universidad Católica de Murcia, 30107 Murcia, Spain; ecuevas@ucam.edu; 2 Departamento de Psicología Social, Facultad de Ciencias de la Salud, Universidad de Alicante, 03690 Alicante, Spain; fina.anton@ua.es

**Keywords:** academic burnout, personality, nursing, education

## Abstract

This study examines academic burnout syndrome and its relation to personal and academic variables among university students in nursing and early childhood education programs in Spain. A total of 606 university students (primary education: 49.7%; nursing: 49.7%) of both sexes (71.5% female) with an average age of 20.68 years (SD = 1.65) participated. An ex post facto retrospective single-group design was planned. The instruments used were the Maslach Burnout Inventory-Student Survey (MBI-SS) and the NEO Five-Factor Inventory (NEO-FFI). Nursing students, who reported more study hours, less sleep, and lower grades, had higher academic burnout scores. Linear regression models were proposed to analyze the relationship between academic burnout, personality, and sociodemographic variables. Nursing students scored higher in emotional exhaustion and lower in cynicism, and they scored higher in neuroticism and openness. Furthermore, 16.1% of the variance in academic burnout was explained by personality variables as well as the degree studied, course year, and study hours. These findings suggest the importance of considering both academic and personality variables in understanding academic burnout in university students.

## 1. Introduction

Burnout syndrome is a psychological phenomenon extensively studied that impacts individuals under high chronic stress, particularly in work environments. First coined by Freudenberger in 1974 [1], it described emotional exhaustion and depersonalization among healthcare professionals. Freudenberger recognized that it was distinct from anxiety, stress, or depression, tentatively naming it burnout, and identified four key variables to differentiate burnout from these constructs: (1) pressure to meet the demands of others, (2) intense competitiveness, (3) the desire to earn more money, and (4) the feeling of being deprived of something one deserves. The concept gained momentum years later through the work of social psychologists Maslach and Jackson in 1981 [2], leading to an extensive scientific literature [3]. They proposed a definition involving three dimensions: emotional exhaustion, depersonalization, and reduced personal accomplishment. Emotional exhaustion is characterized by feeling emotionally drained and fatigued, not due to physical causes like sports exertion. Depersonalization refers to emotional distancing from those one works with, a detachment from aversive experiences. The third dimension, reduced personal accomplishment, encompasses the inevitable feeling of not achieving personal or professional development, not meeting professional goals, or feeling that work is meaningless, which is linked to perceived self-efficacy reduction [4].

This definition and conceptualization of the syndrome was adopted by international research, and it remains the most accepted one today, with slight linguistic nuances. Since then, the concept has expanded widely and is applied to various professions and contexts, including the academic field. Some authors, following up on the original proposal of the syndrome, raised the unquestionable fact that burnout could be observed in other areas, such as the workplace [2,4] and on the sporting [5], academic [6], social [7], marital [8], or even individual planes [9]. Furthermore, the age range in which burnout syndrome was studied was broadened, not only focusing on adults or university students, as it was observed that even school children had burnout [5,10].

Academic burnout syndrome, also known as “educational burnout” or “burnout in education”, is a specific manifestation of burnout that affects professionals in the educational field, such as teachers, researchers, and students. It was in the 1990s that the first studies on burnout in university students appeared, and, in a very recent systematic review [11], the authors conclude that academic burnout is more frequently experienced by students than by teachers, in contrast to the now outdated approach of Maslach and Jakcson [2] of wanting to place burnout solely in the workplace.

Although there is no universally accepted definition, scholars have identified common symptoms that characterize academic burnout. It is characterized by the same symptoms as general burnout (physical and emotional exhaustion, demotivation towards studies and emotional disconnection from the academic environment, and a decrease in the sense of achievement and personal efficacy), but it is associated with specific factors of the academic environment, such as academic pressure to obtain good grades, the intense workload and the lack of resources to meet educational demands, meeting tight deadlines and following an accelerated study pace, comparison with other students and pressure to excel, difficulty in reconciling study with other activities, etc. [12].

Many authors have highlighted that academic burnout can have a negative impact on mental health, academic performance, and satisfaction with higher education [13]. Therefore, some research has focused on studying the quality of life of students and analyzing its relationship with other variables such as lifestyle [14] and the importance of selfcare [15]. Thus, a study regarding psychosomatic aspects associated with burnout in nursing students [16] observed not only problems such as sleep disturbances but even more serious psychological problems such as depression. This result was similar to that obtained one year later with medical students [17], where academic burnout maintained an inverse relationship with the level of mental health of these young people. Recent results also showed how academic burnout in engineering students correlated with other problems such as obsessive–compulsive disorders, depression, phobic anxiety, or paranoid ideation, to name just a few examples [18]. It has been empirically demonstrated that healthcare [17,19,20,21] and teaching [22,23,24] professionals are the most affected by burnout syndrome. These are professions that require dedication, involvement, idealism, service to others, and a personality with a high degree of self-demand and a great tendency to get involved at work [25]. Data from recent studies in nursing have found a high prevalence (40.55%) of emotional exhaustion [26]; in military nursing, various levels (10.5–21.1%) of prevalence of each dimension have been reported [27]. In the case of teachers, Garcia-Carmona et al. [28] found a higher prevalence (28.1–40.3%) of high levels of each dimension, noting that the prevalence of burnout in this sector should be further studied and reported. Both at the professional and pre-professional levels, the demands of these professions can trigger stress responses, anxiety, and emotional exhaustion, even more so at younger ages.

To address this problem, it is essential to identify the underlying causes and develop appropriate prevention and support strategies to promote the well-being of university students and ensure a healthy academic environment. The risk factors for academic burnout are multiple and can be of a personal, organizational, or occupational nature [29,30]. Personal factors include personality, coping style, and physical and mental health. Organizational factors include workload, academic pressure, lack of control, lack of social support, and insecurity and uncertainty about the future job market. Occupational factors include lack of meaning in work, lack of recognition, and dissatisfaction with a professional career.

Linking with personality, the need to attend to the psychological profile of the subject if it was to be addressed from a psychopathological perspective had already long ago been pointed out [31]. Therefore, in this work, in addition to analyzing academic burnout syndrome, we aimed to observe the relationship that the problem has with the personality of the students. To do this, we investigated the differences that can potentially be found by taking into account the degree studied—starting from the hypothesis that perhaps nursing students are the ones who present the highest levels of burnout, following recent findings that up to a third of nursing students could suffer from it [32]—or which showed how the weakness of the variables related to the less resistant personality caused a higher propensity to develop the syndrome [33].

We are interested in understanding, if this hypothesis is confirmed, what the reasons may be for why some university careers cause more burnout than others, and for this it was essential to verify whether the personality characteristics, in their configuration as a pattern of functioning, could explain this reality. Likewise, we are interested in determining the relationship between academic variables, such as the degree studied (comparing in this case nursing and early childhood education students), the academic year, the hours dedicated to studying, and the students’ average grade, with academic burnout. 

Therefore, the main objectives of this study were (1) to examine the differences in academic burnout levels among university students based on their degree, academic year, and weekly study hours; and (2) to investigate the relationship between university student personality characteristics and academic burnout.

Based on the scientific literature, the hypotheses were (1) higher levels of academic burnout are expected in nursing students; and (2) academic burnout is positively related to neuroticism and negatively related to the rest of the personality traits.

## 2. Materials and Methods

### 2.1. Sample

The present study was conducted with a random sample of university students, all of whom were voluntarily recruited and selected at random. The inclusion criteria included active enrollment in the academic programs of nursing or early childhood education, as well as possessing Spanish nationality. The exclusion criterion was that the participants’ age was over 26 years old.

Meeting all of these criteria, a sample of 606 Caucasian university students with an average age of 20.68 years and a standard deviation of 1.65 years were included. The sample comprised 71.5% females, and regarding their fields of study, 49.7% of the students were enrolled in nursing and the remaining 50.3% in early childhood education. The distribution of students per year was balanced, with a slight predominance of students in the third year (33.6%), followed by the fourth (25%), second (21.9%), and first (19.5%). As for the average grade of the students, the most frequent was B, with 72% of the subjects obtaining average grades in the range of 7–8.9 points. This was followed by the pass rate (20.5%), followed by outstanding (5.7%), and finally, with a very low percentage, the failure rate (1.8%). Regarding marital status, most of the participants were single (96.1%), while 2.9% were married and 0.3% separated. In addition, only 3% of the students had dependent children. The participants spent 7.49 h per week studying (SD = 6.485) and slept approximately 7.2 h per day (SD = 1.930). 

### 2.2. Measures

An ad hoc questionnaire was employed to assess sociodemographic variables necessary to describe the sample. This questionnaire inquired about age, sex, marital status, dependents, hours dedicated to study and sleep, and the average grade from the previous academic year.

Regarding academic burnout, it was examined using the Maslach Burnout Inventory-Student Survey (MBI-SS) [34], adapted and validated for the Spanish population [35]. This questionnaire consists of 15 items rated on a Likert scale from 0 to 6, where the lowest score (0) corresponds to “never” and the highest score (6) to “every day”. Additionally, this questionnaire has three subscales: emotional exhaustion, cynicism, and efficacy. According to this questionnaire, high scores in the first two factors and low scores in the last are indicative of higher levels of academic burnout. The internal consistency of the MBI-SS in this study was 0.716. Regarding the reliability of the questionnaire, Cronbach’s Alpha of 0.87 was obtained for the emotional exhaustion dimension, 0.78 for the cynicism dimension, and 0.75 for the efficacy dimension, with an Alpha of 0.72 for the total score. These data are similar to (and even higher than) those obtained by the authors in the original version: emotional exhaustion: 0.74, cynicism: 0.79, and efficacy: 0.76 [35].

Personality was assessed using the NEO Five-Factor Inventory (NEO-FFI) [36] in its Spanish version [37]. This questionnaire comprises 60 items rated on a Likert scale with five response options (ranging from strongly disagree to strongly agree) and presents five subscales: neuroticism, extraversion, openness, agreeableness, and conscientiousness. In the present study, internal consistency reliability ranged from 0.69 to 0.82 (neuroticism = 0.82; extraversion = 0.80; openness = 0.73; agreeableness = 0.69; and conscientiousness = 0.79; total score = 0.75), values similar to those reported by the authors in the original version (Alpha range = 0.71–0.86) [37]. 

### 2.3. Procedure

To analyze the relationships between academic burnout, personality traits, and sociodemographic variables, a retrospective ex post facto single-group design [38] was carried out. Other academic variables such as the degree studied and the average grade obtained by the students the previous year were also analyzed.

Upon obtaining the approval of the University’s Research Ethics Committee (CEO1201, 2023), the deans of the faculties of education and nursing were contacted to explain the purpose of the study and to arrange the most suitable dates for administering the questionnaires. In the classroom context, the standardized procedure carried out to ensure uniformity in the instructions to all participants was explained. Furthermore, prior to completing the booklet, informed consent was obtained from all subjects involved in the study. Assurance of anonymity and confidentiality of the data provided was given to all participating individuals, in addition to having the continuous presence and guidance of the research team, who supervised the sessions in which the measurement instruments were administered for individual completion, lasting approximately 50 min.

### 2.4. Data Analysis

In relation to the statistical analyses, a study of basic descriptors and frequency of all variables was carried out. In addition, several tests were used to observe the differences between subgroups and establish their different profiles. In the case of continuous variables, the Student’s *t*-test was used, and for categorical variables, Chi-squared. In relation to the analysis of academic burnout and personality, the Student’s *t*-test was performed to observe the differences between groups. Cohen’s d was used to establish the magnitude of the differences between two groups (large effect magnitude from 0.8; moderate effect magnitude between 0.5 and 0.79; small effect between 0.2 and 0.49) [39]. Next, two linear regression models were proposed, generating the corresponding mean scatter plots to observe the data. The significance level used in the analyses was 5% (*α* = 0.05). Both for the analysis and for the graphs, they were performed using the statistical program Jamovi, 2.3.21.0.

## 3. Results

### Preliminary Analysis

Regarding the sociodemographic differences between the subgroups, differences were observed in the variables of age, hours of study, and hours of sleep. Examining the age of the participants, statistically significant differences were perceived with a medium-high effect size (*t*604 = 3.72, *p* < 0.001, *d* = 0.302); regarding the variable of study hours, statistically significant differences were observed with a high effect size (*t*604 = −6.15, *p* < 0.001, *d* = −0.500); regarding the sleep hours variable, statistically significant differences were also noted with a medium-low effect size (*t*604 = 2.26, *p* = 0.024, *d* = 0.184). Table 1 illustrates the magnitude of these differences, indicating that nursing students were younger, studied more hours, and slept less. Furthermore, statistically significant differences between the subgroups were also observed in other variables, such as the sex of the participants (χ^2^= 10.4, *p* < 0.001). However, no differences were found in marital status (χ^2^ = 1.01, *p* = 0.316) and number of children (χ^2^ = 1.04, *p* = 0.308). As shown in Table 2, 77.4% of the nursing students were female compared to 65.6% of female students in early childhood education.

Furthermore, differences were also observed in the grades achieved. Early childhood education students obtained higher grades compared to nursing students. As depicted in Table 2, both ‘excellent’ and ‘very good’ grades occurred more frequently in early childhood education students: 65.2% and 55.3%, respectively. In contrast, nursing students had a higher frequency of lower scores, and though the ‘fail’ response could not be analyzed due to its low occurrence in both groups, it should be noted that 68.4% of the students who scored a ‘pass’ were from the nursing program.

Since the study’s objective was to analyze the differences in academic burnout and personality among students from different fields, the Student’s *t*-test was conducted to assess the observed differences between the two subgroups. As shown in Table 3, differences were found between the total score of academic burnout and some of its factors (exhaustion and cynicism). Notably, in the academic burnout data, the effect size for the exhaustion factor was very high (*t*604 = −9.352, *p* < 0.001, *d* = 0.760), in contrast to the efficacy factor, where no statistically significant differences were observed (*t*604 = 0.889, *p* = 0.374, *d* = 0.072).

Continuing with the data analysis from Table 3, statistically significant differences were only observed in the following personality variables: neuroticism and openness. For neuroticism, a difference with a low effect size was noted (*t*604 = −2.121, *p* = 0.034, *d* = −0.172), and for openness, a medium effect size (*t*604 = −2.433, *p* = 0.015, *d* = 0.198). These findings suggest that nursing students scored higher in emotional exhaustion and lower in cynicism. However, no differences were observed in efficacy. In terms of personality, nursing students scored higher in neuroticism and openness; no differences were observed in the other scores.

Comparing the relationship between these variables, statistically significant differences were observed between personality factors and academic burnout. Specifically, a linear regression model was proposed between neuroticism, agreeableness, and conscientiousness with a low effect size (F3, 602 = 24.6, *p* < 0.001, *d* = 0.109). These data suggest that both scores in conscientiousness and neuroticism are directly related to levels of academic burnout, whereas higher scores in agreeableness lead to lower academic burnout scores. However, the most comprehensive model includes the personality variables (described above) along with the degree studied, course year, and study hours (F8, 597 = 14.4, *p* < 0.001, *d* = 0.161). This last analysis accounts for 16.1% of the variance explained in academic burnout.

**Figure 1:**
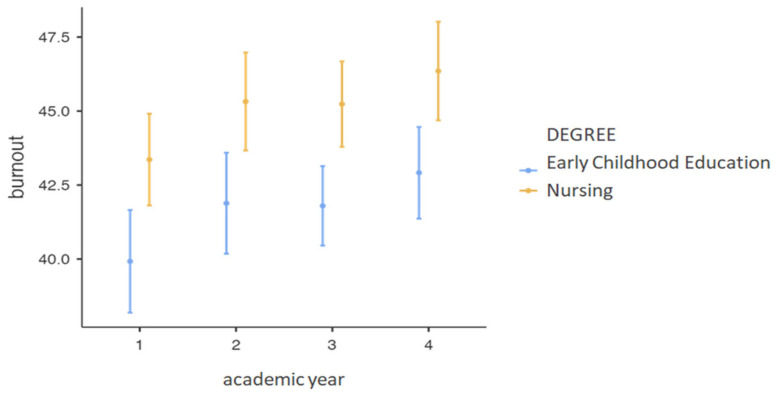
Relationship between Burnout, Course Year, and Degree.

**Figure 2:**
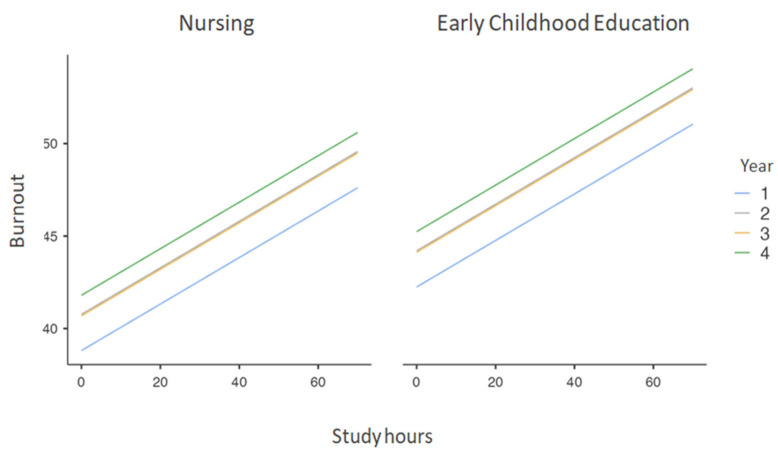
Relationship between Burnout, Study Hours, and Degree.

These findings are also reflected in Figure 1 and Figure 2, where it can be observed that nursing students scored higher in academic burnout as they progressed through their courses, with a notable difference between the first and fourth years. This difference between degrees is also evident in the study hours and their relationship with academic burnout.

## 4. Discussion

This study aims to be a preliminary approach to the reality of university students experiencing academic burnout and the relationship this syndrome may have with sociodemographic and academic variables, as well as personality traits. It is true that the comparison made does not provide definitive data on some of the hypotheses presented, but it is interesting to note that nursing students show a higher level of academic burnout, as indicated by previous works [33]. These studies also established certain connections between the syndrome and personality traits, with neuroticism being positively correlated and agreeableness negatively correlated in our case.

The fact that nursing students clearly present a younger age profile, with greater dedication to study and consequently fewer hours dedicated to sleep, aligns with some contributions from studies conducted with other student population groups regarding sleep disorders [16], increased commitment to studies [40], and improvement of developed habits [41]. However, undoubtedly the most interesting aspect is the confirmation that the study of personality, as initially related to the syndrome [31], continues to be an essential aspect if we wish to understand the reality of academic burnout when it affects, in this case, students in general, or nursing students in particular. Only through this approach can we delve deeper into improving the mental health of our students with effective programmatic actions [11,17,18].

Regarding the influence of the degree on the students’ experience of academic burnout (Hypothesis 1), the highest levels were expected in the sample of nursing students according to the scientific evidence. Indeed, higher rates of total academic burnout were observed in nursing students than in early childhood education students. This hypothesis was confirmed. 

Forty-three percent of students in the nursing degree are “burned out”, i.e., they register high levels of academic burnout, compared to 23% of “burned out” students in early childhood education. The findings of this study are consistent with evidence that nursing students, given the academic and clinical demands, are particularly at risk of experiencing higher levels of academic burnout. Several studies [42,43,44,45,46,47] have also reported similar results, indicating high levels of emotional exhaustion in healthcare students. All of these disparities may reflect differences in curricular structures, in the nature of professional practices, and, potentially, in the coping strategies and social support available to these students. The findings highlight the importance of recognizing and addressing academic burnout in university student populations, with particular attention to differences between disciplines. Analyzing the curricula of the degree programs, we found that it is in the final years that curricular placements are concentrated; the different nature and demands of these—in the area of health and in the area of education—must be a variable to be considered when trying to understand and explain the differences in the relationship between academic year and academic burnout in the different degree programs.

With regard to the relationship between personality traits, it was expected that academic burnout is positively related to neuroticism and negatively related to the rest of the personality traits (Hypothesis 2). In the light of the results obtained, the hypothesis was partially confirmed. Research on the connection between academic burnout and personality is based on the idea that individual traits such as personality affect the experience of burnout. This would imply that people may be more or less prone to academic burnout depending on their personality traits. Neuroticism was a positive explanatory variable for academic burnout. This finding is consistent with all the research reviewed on the subject, which unanimously shows a relationship between neuroticism and academic burnout [48,49,50,51]. In terms of education policy and student well-being, these results underline the need for a more holistic approach to student development, one that considers personality as an integral factor in the educational experience. Intervention and well-being programs could be more effective if they were personalized to suit different personality types, thus helping students not only to succeed academically, but also to thrive during their time at university.

This work is not without limitations. A first limitation is the lack of homogeneity in terms of the sex variable, since the number of female students (71.5%) is much higher than that of male students (28.5%). This is due to the fact that the degrees taken by the participants (nursing and early childhood education) are traditionally professions developed to a greater extent by women. This circumstance makes it necessary to make comparisons in terms of gender with caution.

Continuing with the sample, the students interviewed were limited to a single university center, and both the institutional context and educational policies may have influenced the results that were not contemplated a priori. Therefore, it would be advisable to have a university population from different centers and autonomous communities to be able to establish representative and generalizable conclusions.

The cross-sectional design planned in the doctoral thesis presented here, although it provides a valuable snapshot of the state of academic burnout at a specific time, limits the ability to establish causal relationships between the variables examined. Therefore, it is proposed for future research to homogenize the groups in order to be able to carry out comparative analyses.

## 5. Conclusions

Several conclusions can be drawn from the findings of the conducted study:

It is important to conduct research that focuses on the personality of the student and its relationship with academic burnout syndrome from different theoretical perspectives that link it with other variables, such as sociodemographic ones.

It would also be interesting to introduce new variables related to maintained habits, such as physical activity, diet, and hours dedicated to rest, among others.

There is a need to increase the number of university degrees analyzed to verify whether there are indeed trends that link academic burnout more evidently in some studies than in others.

Consideration should be given to the psychological variables of the student, both those related to their personality and those more specific to mental health, when developing prevention programs for the syndrome.

For future studies, it would be advisable to increase the number of sociodemographic variables, as this could aid in understanding the variation in behavior among different students with academic burnout.

The robustness of the theoretical proposal of this study was maintained, confirming the appropriateness of analyzing academic burnout syndrome and personality profiles in an attempt to optimally understand this psychological problem, and it is evident that academic burnout remains associated with problems of the neurotic spectrum (neuroticism).

## Figures and Tables

**Table 1:** Sample Description by Age, Study Hours, and Sleep Hours.

		Mean	*SD*	Min	Max	*t*	*p*	*d*
Age	E.C. Educ.	20.93	1.53	18.0	25.0	3.72	<0.001 *	0.302
	Nursing	20.44	1.73	18.0	25.0			
Study hs	E.C. Educ.	6.87	6.79	0.0	68.0	−6.15	<0.001 *	0.500
	Nursing	10.83	8.90	0.0	70.0			
Sleep hs	E.C. Educ.	7.25	2.01	0.0	12.0	2.26	0.024 *	0.184
	Nursing	6.94	1.18	0.0	10.0			

* *p* significant value.

**Table 2:** Sample description according to the average grade obtained in the previous course.

Average Grade	E.C. Education		Nursing		Statistics	*p*
D	1	(33.3%)	2	(66.7%)	25.7	<0.001 *
C	43	(31.6%)	93	(68.4%)		
B	242	(55.3%)	196	(44.7%)		
A	15	(65.2%)	8	(34.8%)		

* *p* significant value.

**Table 3:** Differences in Burnout and Personality in Nursing and Early Childhood Education Students.

		Mean	SD	Min	Max	*t*	*p*	*d*
Burnout	E.C. Educ.	41.58	7.82	18.00	65.0	−5.07	<0.001 *	−0.412
	Nursing	45.17	9.55	23.00	90.0			
Emotional exhaustion	E.C. Educ.	9.69	5.62	0.00	27.0	−9.35	<0.001 *	−0.760
	Nursing	14.28	6.43	1.00	30.0			
Cynicism	E.C. Educ.	4.55	3.98	0	19	1.92	0.056	0.156
	Nursing	3.90	4.37	0	24			
Efficacy	E.C. Educ.	27.34	4.65	11.00	36.0	0.89	0.374	0.072
	Nursing	26.99	4.88	11.00	36.0			
Neuroticism	E.C. Educ.	19.95	8.18	0.00	47.0	−2.12	0.034 *	0.072
	Nursing	21.36	8.20	1.00	43.0			
Extraversion	E.C. Educ.	34.21	6.91	15.00	48.0	0.45	0.654	0.364
	Nursing	33.96	6.80	9.00	48.0			
Openness	E.C. Educ.	27.91	6.35	13.00	44.0	−2.43	0.015 *	0.198
	Nursing	29.16	6.31	12.00	48.0			
Agreeableness	E.C. Educ.	31.43	5.85	11.00	46.0	−0.81	0.419	−0.066
	Nursing	31.82	5.86	12.00	46.0			
Conscientious	E.C. Educ.	31.58	6.56	15.00	48.0	−0.17	0.865	−0.014
	Nursing	31.67	6.59	11.00	47.0			

* *p* significant value.

## Data Availability

Not applicable.
